# Pannexin 1 inhibition delays maturation and improves development of *Bos taurus* oocytes

**DOI:** 10.1186/s13048-020-00704-w

**Published:** 2020-08-24

**Authors:** Zachary Timothy Dye, Lauren Virginia Rutledge, Silvia Penuela, Paul William Dyce

**Affiliations:** 1 Department of Animal Sciences, Auburn University, CASIC Building, 559 Devall Drive, Auburn, AL 36849 USA; 2 Department of Anatomy and Cell Biology, Western University, London, Ontario Canada

**Keywords:** Cumulus, Developmental biology, Early development, In vitro maturation (IVM), In vitro fertilization (IVF), Oocyte development, Oocyte maturation

## Abstract

**Background:**

Intercellular exchange between the oocyte and its surrounding cells within the follicular environment is critical for oocyte maturation and subsequent development. In vertebrates this exchange is facilitated through gap junctions formed by connexin membrane proteins. Another family of membrane proteins called pannexins are able to form single membrane channels that allow cellular exchanges with the extracellular environment. The most ubiquitously expressed and studied member, pannexin 1 (PANX1), has yet to be described thoroughly in female reproductive tissues or functionally studied during oocyte maturation. Here, we look into the expression of pannexin 1 in bovine cumulus-oocyte complexes (COCs), as well as, its potential role in oocyte maturation and development.

**Results:**

We show that pannexin 1 is expressed in bovine COCs and that the expression of PANX1 was significantly lower in COCs isolated from large antral follicles (> 5 mm) compared to those isolated from small antral follicles (< 2 mm). Supporting this we also found lower expression of PANX1 in oocytes with higher developmental potential when compared to oocytes with lower developmental potential. We further found that PANX1 channel inhibition during in vitro maturation resulted in temporarily delayed meiotic maturation and improved in vitro developmental outcomes while decreasing intercellular reactive oxygen species.

**Conclusions:**

These data suggests PANX1 is differentially expressed at a critical stage of follicular development when oocytes are acquiring developmental competence, and may play a role in the timing of oocyte maturation.

## Background

During folliculogenesis, communication between the oocyte and its surrounding cells is critical for proper maturation of the oocyte following ovulation. Intercellular exchange between the oocyte and follicular cells is facilitated by gap junctional communication [1]. Gap junctions, in mammals, are formed by the family of membrane proteins called connexins [2]. Connexins facilitate the exchange of nutrients, metabolites, and secondary messengers between neighboring cells [3]. Connexins have also been shown to play an important role in oocyte maturation and development, with the loss or reduction of connexin 43 having negative impacts on fertility in multiple species [4–7].

A more recently identified family of membrane proteins named pannexins (PANX), with limited sequence homology to the invertebrate gap junction family of innexin proteins, was discovered by Panchin et al. [8]. The pannexin family of glycoproteins consists of three members pannexin 1, pannexin 2, and pannexin 3 (PANX1, PANX2, and PANX3). All three are predicted to be tetra-spanning membrane proteins with the amino and carboxy terminal ends present in the cytoplasm, as well as two extracellular loops that contain two cysteine residues each and a N-glycosylation site [9, 10]. Though initially thought to form gap junctions like connexins, pannexins have also been shown to be structurally different and form single membrane channels that provide a pathway for molecular exchanges between the cell’s cytoplasm and extracellular environment [10–12].

PANX1, the most extensively studied of the pannexins, has been found to be ubiquitously expressed in many tissue types. In human tissues, PANX1 is expressed in the heart, skeletal muscle, testis, ovary, brain, placenta, thymus, prostate, and small intestine [13]. PANX1 is also highly expressed in central nervous tissues and neuronal cells in mice [13, 14]. Though shown to be expressed in both female and male glands, the role of PANX1 in reproductive cells remains very limited. However, PANX1 has been recently shown to play a role in oocyte health in humans [15]. A mutation in PANX1 in humans has been described that results in a loss of control of cellular functioning [15]. Oocytes having the mutation, following retrieval, released more adenosine 5′-triphosphate (ATP) to the extracellular space and degenerated. The mutation appeared to affect maturation potential in the oocytes as they were able to collect very few mature oocytes with the majority being immature and all degenerating at or very shortly after fertilization [15].

Functionally, Panx1 has been implicated in many physiological processes including the removal of apoptotic cells [16, 17], inflammation [18], viral infection [19], ischemia [20], and neurological functions [21, 22]. One major role involves facilitating the release of ATP into the extracellular environment [11, 23, 24]. ATP release is achieved after pannexon channel activation by way of purinergic receptors or cytoplasmic calcium [25–27]. PANX1 has also been linked to functional roles in vasodilation and constriction, taste sensation, and HIV infection [19, 28, 29]. Panx1 appears to have different functional roles based upon stimulation type such as voltage activation or truncation; leading to more potential roles for the channel [30]. It is important to note that while PANX1 expression has been found in the gonads of both males and females, its biological role is not clearly defined as Panx1 knockout mice appear fertile [31].

While the specific role of pannexins in oocyte maturation remains undefined, connexins and innexins have been clearly implicated. It has been shown that the resumption of meiotic maturation out of prophase-arrest is inhibited in cattle and mice if connexin channels are inhibited or knocked-out [32, 33]. Furthermore, innexin gap junction channels are required for proper meiotic maturation to occur in *C. elegans* [34]. Mechanistically, gap junctional communication in bovine cumulus-oocyte complexes (COCs) facilitates the decline in cyclic adenosine 3′,5′-monophosphate (cAMP) which is one proposed mechanism of oocyte meiotic resumption [35, 36].

To date there is little information regarding the expression and functioning of pannexin channels within ovarian tissues. We hypothesize that PANX1 plays a role during oocyte maturation. We investigated the expression of PANX1 in bovine COCs and its potential role in oocyte maturation and early embryo development, following in vitro fertilization (IVF).

## Results

### Pannexin 1 expression in bovine cumulus-oocyte complexes

**Fig. 1:**
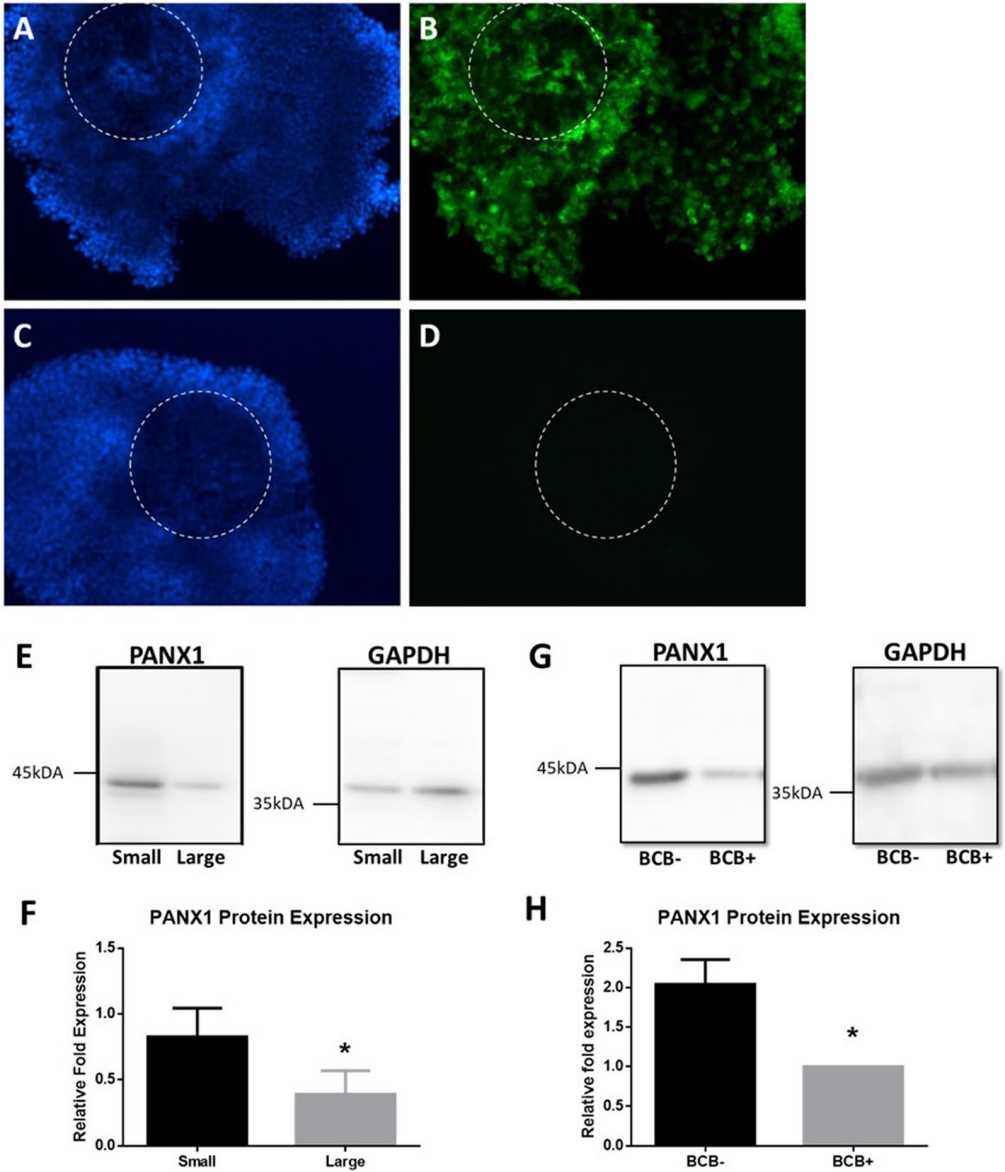
Pannexin1 expression in Bovine Cumulus-Oocyte Complexes, During Different Follicular Stages, and Varying Stages of Oocyte Developmental Competence. **a** Representative immunofluorescent images of bovine COCs stained with Hoechst33342 (blue, **a**) and labeled with PANX1 antibody (green, **b**). **c** A representative image depicting a bovine COC stained with Hoechst33342 and stained with secondary antibody only (**d**). Oocyte outlined with dashed circle. Bars = 100 μm. **e** Representative blot images depicting the levels of PANX1 expression in granulosa cells isolated from small (< 2 mm) and large (> 5 mm) antral follicles. GAPDH was used as a loading control. **f** Densitometry results showing the expression level of PANX1 in granulosa cells isolated from small and large antral follicles. **g** Representative blot images depicting the levels of PANX1 in BCB- and BCB+ COCs. **h** Densitometry results showing the expression of PANX1 in BCB- and BCB+ groups of COCs. *Denotes a significant difference, *p* < 0.05. Error bars are ± SD from mean

PANX1 immunoflourescent expression was seen in bovine COCs. PANX1 is localized in cumulus cells with a ubiquitous expression pattern (Fig. 1a-d). PANX1 expression was different at different stages of follicular development or in COCs with differing developmental potentials. The level of protein expression was measured using western blotting to compare the expression of PANX1 in granulosa cells isolated from small antral follicles (< 2 mm) to those from large antral follicles (> 5 mm). Granulosa isolated from small follicles had a significantly higher expression of PANX1 (0.827 ± 0.217) compared to granulosa isolated from large follicles (0.394 ± 0.176, *p* = 0.0211)(Fig. 1e,f). Oocyte quality was categorized by staining with BCB. COCs containing oocytes that were BCB negative had significantly higher expression of PANX1 when compared to BCB positive oocyte containing COCs (2.043 ± 0.314 and 1.000 ± 0.0 respectively, *p* = 0.0045) (Fig. 1g,h).

### Inhibiting the pannexin 1 channel

**Fig. 2:**
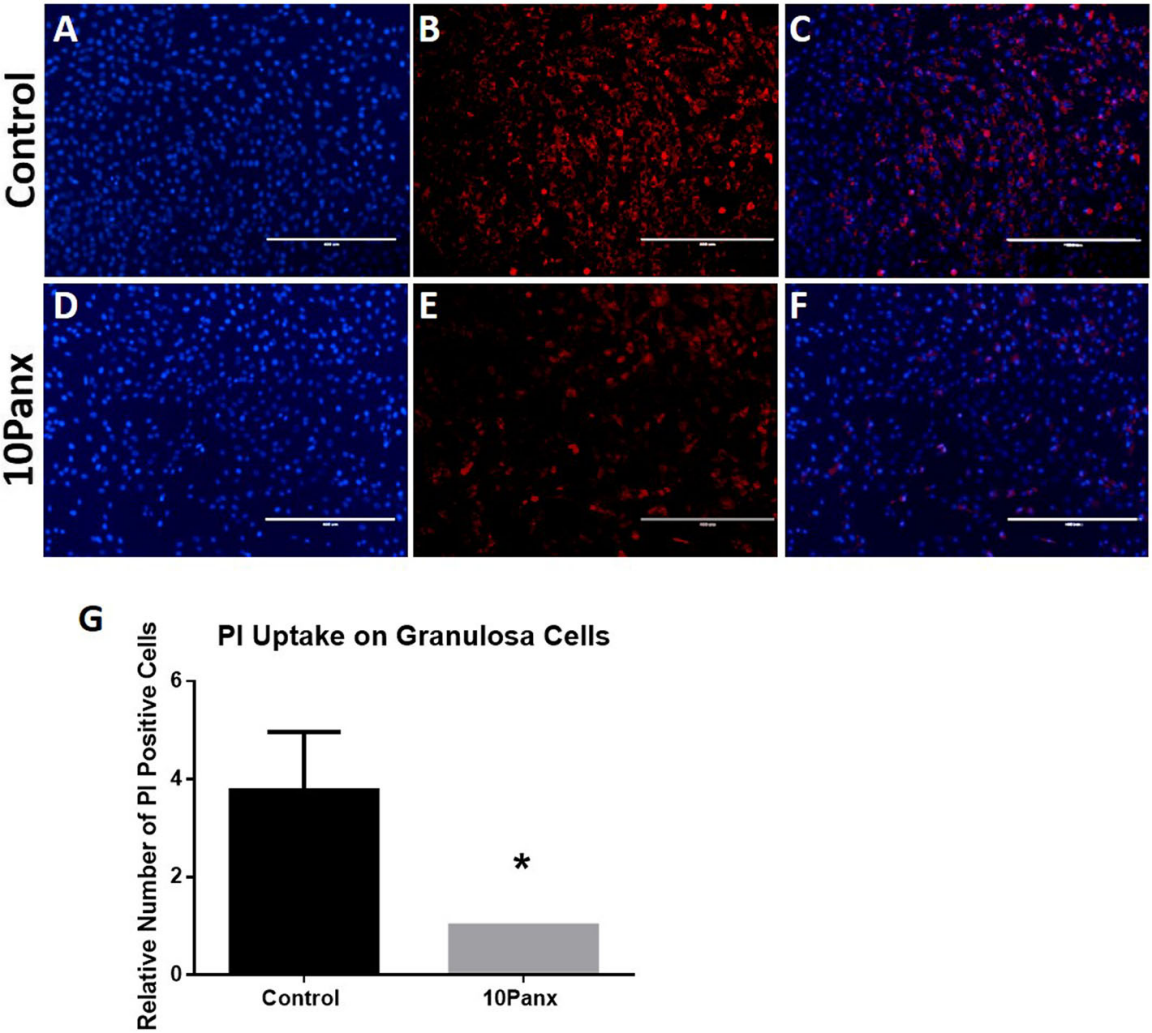
Effect of Inhibiting the Pannexin 1 Channel on Hemi-channel Function. Representative images depicting PI dye uptake (red) by untreated granulosa cells (**b**) and granulosa cells treated with 10Panx (**e**). Cells were nuclear stained with Hoechst33342 (blue, **a** & **d**) and overlayed (**c** & **f**). Bars = 400 μm. **g** The relative number of PI positive cells in control or 10Panx treated granulosa cells. *Denotes a significant difference, *p* < 0.05. Error bars are ± SD from mean

In order to establish that the 10Panx mimetic peptide inhibitor was indeed blocking PANX1 channels, a dye uptake study was performed using the hemichannel permeable dye PI. Cultured granulosa cells that had been incubated for 30 min with or without 10Panx were measured for PI uptake. A significant decrease in dye uptake was found in 10Panx treated cells compared to the control cells (*p* = 0.0143). The relative fold proportion of PI positive cells was greater in the control with this group having 1.846 ± 0.353 fold more positive cells than the 10Panx group (Fig. 2, Figure shows data that was non-transformed ratios, log transformed data was used for statistics).

### Effect of pannexin 1 inhibition on in vitro matured cumulus-oocyte complexes

#### Cumulus expansion

**Fig. 3:**
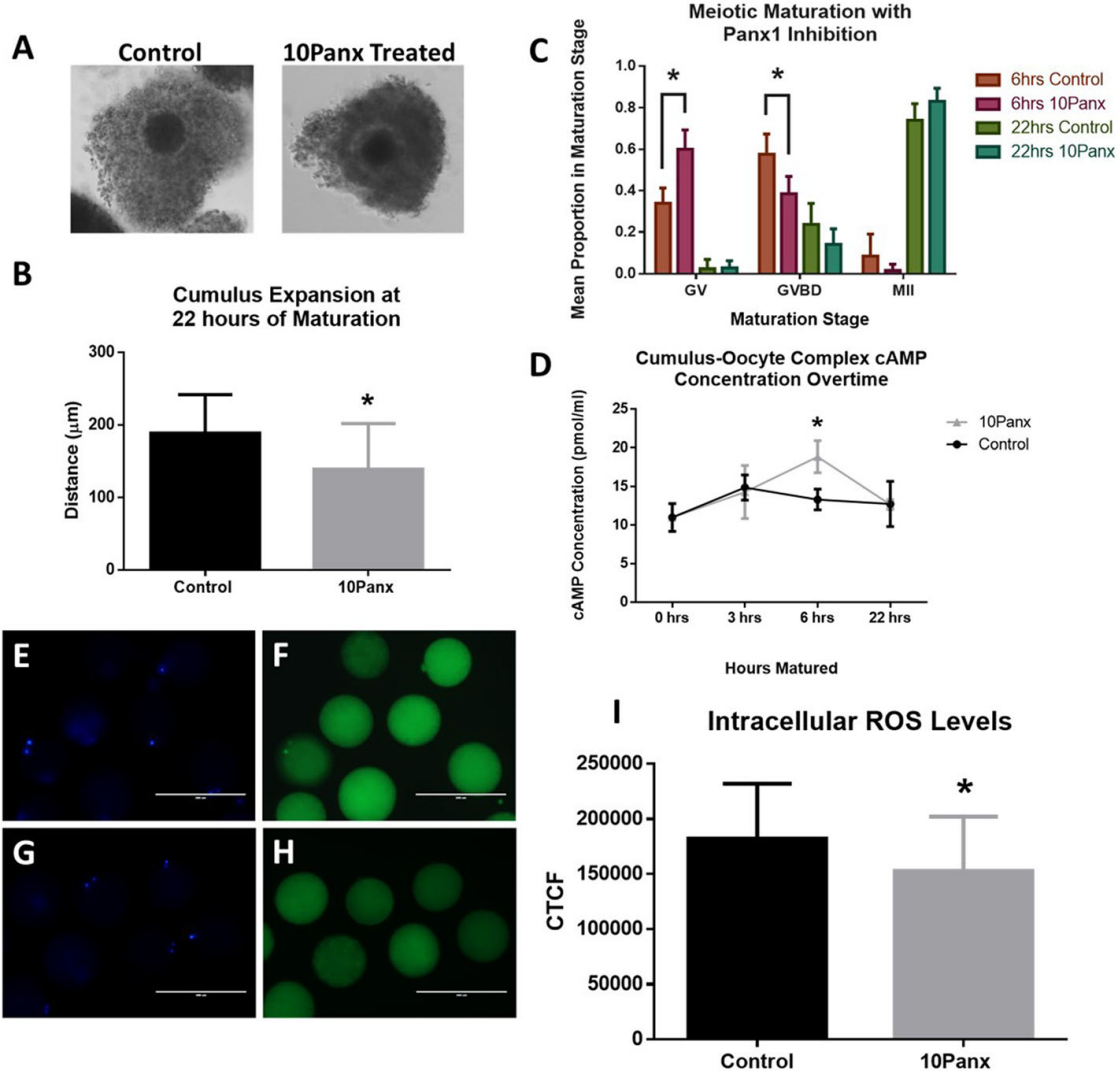
ROS and Maturation Effects of Pannexin 1 Inhibition. **a** Representative images of cumulus expansion after 22 h of IVM in control COCs and PANX1 inhibited COCs. Magnification was 20x. **b** Cumulus expansion in the control and 10Panx treated groups. **c** Meiotic maturation proportions of oocytes at the GV, GVBD, and MII stages after 6 h and 22 h of IVM with or without PANX1 inhibition. **d** The average icAMP concentration of COCs treated with or without 10Panx at 0, 3, 6, and 22 h of IVM. *Denotes a significant difference, *p* < 0.05. Error bars are ± SD from mean. Representative images depicting the staining intensity of DCFH-DA showing levels of free radicals within the oocyte after 22 h of IVM without (**f**) or with (**h**) PANX1 inhibition. Cells were nuclear stained with Hoechst33342 (blue, **e** & **g**). Bars = 200 μm. **i** Quantitation of the staining intensity of DCFH-DA using CTCF. *Denotes a significant difference, *p* < 0.05. Error bars are ± SD from mean

To study the initial effects of PANX1 inhibition on the in vitro maturation of COCs, we measured the expansion of cumulus cells in COCs after a maturation time of 22 h with or without 10Panx supplementation. The 10Panx treated COCs were found to have less cumulus expansion compared to the vehicle only control group (152.600 ± 30.460 μm and 193.200 ± 13.890 μm respectively, *p* = 0.0264)(Fig. 3a, b).

#### Oocyte meiotic maturation

We next looked into the effects that the inhibition of PANX1 had on the stage of DNA maturation after 6 and 22 h of maturation. COCs were matured in vitro, stripped of cumulus cells, stained with Hoechst33342, and DNA maturation stage was observed under a fluorescence microscope. DNA maturation stages were categorized into GV, GVBD, and MII stages. Following 6 h of in vitro maturation (IVM), COCs treated with 10Panx had a significantly higher proportion (0.600 ± 0.094) of DNA in the GV stage compared to the control group (0.340 ± 0.073, *p* < 0.0001). There was a significantly higher proportion of oocytes in the GVBD stage after 6 h in the control group compared to the 10Panx treated group (0.576 ± 0.097 and 0.385 ± 0.083 respectively, *p* < 0.0001). The proportion of COCs at the MII stage after 6 h was not significantly different between the 10Panx and control groups (0.015 ± 0.030 and 0.084 ± 0.107 respectively). After 22 h of maturation, the proportion of COCs was no longer significantly different between 10Panx treated and control groups in the GV stage (0.027 ± 0.034 and 0.023 ± 0.046 respectively). There was also no significant difference between the proportions of 10Panx treated and control oocytes in the GVBD (0.140 ± 0.075 and 0.237 ± 0.102 respectively) or MII (0.833 ± 0.061 and 0.741 ± 0.080 respectively) stages after 22 h of maturation (Fig. 3c).

#### Intercellular cyclic adenosine monophosphate concentration of cumulus-oocyte complexes

To further understand the functional role PANX1 has in the bovine COC during maturation, we studied the icAMP levels of intact COCs at various time points during maturation. COCs were matured in vitro with or without 10Panx (100 μM) for 3, 6, or 22 h and were removed, washed, and snap frozen. This was followed by a direct cAMP ELISA to measure icAMP. After 3 h of maturation, the COC icAMP concentration was not significantly different between the 10Panx treated and control groups (14.330 ± 3.434 pmol/ml and 14.910 ± 1.625 pmol/ml respectively, *p* > 0.05). After 6 h of maturation, the COC icAMP concentration was significantly higher in the 10Panx treated group (18.880 ± 2.052 pmol/ml) versus the vehicle only control (13.350 ± 1.345 pmol/ml, *p* = 0.0175). After 22 h of maturation, the COC icAMP was no longer significantly different between the 10Panx treated and control groups (12.690 ± 0.646 pmol/ml and 12.770 ± 2.928 pmol/ml respectively, *p* > 0.05)(Fig. 3d).

### PANX1 inhibition and early embryonic development

**Fig. 4:**
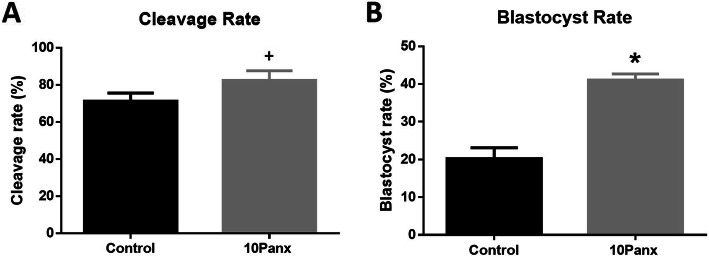
Embryo Development Post Pannexin 1 Inhibition. **a** Cleavage rate at day 2 post IVF with or without 10Panx treatment during IVM. **b** Blastocyst rate at day 7 post IVF with or without 10Panx treatment during IVM. *Denotes a significant difference, *p* < 0.05. +Denotes a significant trend, *p* < 0.055. Error bars are ± SD from mean

To study the effects of PANX1 inhibition during maturation on future preimplantation developmental potential, COCs were isolated from abattoir sourced ovaries, washed, matured in vitro with or without 10Panx supplementation. Matured COCs were then fertilized and cultured in vitro to the blastocyst stage. The embryonic cleavage rate contained a significant trend with the 10Panx treated COCs having higher cleavage (82.420±5.201%) compared to the untreated control COCs (71.240±4.391%, *p* = 0.0529). This was followed by a significantly higher blastocyst rate in 10Panx treated fertilized COCs (41.080±1.599%) compared to untreated fertilized COCs (20.210±2.906%, *p* = 0.0005) (Fig. 4, Figure shows data that was non-transformed percentages, arcsine transformed data was used for statistics).

### PANX1 inhibition and intra-oocyte ROS levels

In order to measure the levels of free radicals in the oocytes following maturation in the presence of 10Panx we compared the DCFH-DA staining level in oocytes following treatment to untreated controls (Fig. 3e-h). The level of reactive oxygen species (ROS) in the oocytes cultured in the presence of 10Panx was found to be significantly lower (144,418 ± 23,134) when compared to the untreated vehicle only controls (177,665 ± 32,725, *p* = 0.0486)(Fig. 3i).

## Discussion

In this study, we describe the expression of PANX1 in bovine oocyte cumulus cells. Interestingly, the expression is differential with higher expression in smaller antral follicles when compared to larger antral follicles. This is consistent with our findings that cumulus cells from BCB+ oocytes have a lower expression level of PANX1 when compared to less developmentally competent BCB- oocytes. BCB staining is used to determine glucose-6-phosphate dehydrogenase (G6pDH) activity, with more developmentally advanced and competent bovine oocytes staining positive [37]. This suggests that during folliculogenesis, and the development of competence in oocytes, the expression of PANX1 decreases in vivo. This is consistent with the findings of Sang et al. who described a mutation in humans that resulted in a loss of control of PANX1 cellular functioning [15]. In that case oocytes having the mutation released more ATP to the extracellular space and following retrieval degenerated. One described mutation appeared to affect maturation potential in the recovered oocytes as very few mature oocytes were obtained with the majority being immature and all degenerating at or very shortly after fertilization [15]. Ours and their findings suggest an important role for PANX1 during oocyte maturation.

In order to explore this further we studied the maturation rate of oocytes exposed to the PANX1 inhibitor 10Panx [38]. We initially found that treatment of oocytes with 10Panx following maturation for 22 h did not change the number of oocytes reaching the MII stage. However, when we looked at the stage of maturation at 6 h we found a significantly higher number of treated oocytes were at the GV stage. These results suggest that the inhibition of the PANX1 hemichannel effectively delays oocyte maturation. Moreover, following exposure to 10Panx the oocytes had higher cleavage and blastocyst rates when compared to untreated controls. This is consistent with others showing that delaying maturation effectively improves the developmental competence of aspirated oocytes. Delaying meiotic maturation has been shown to improve developmental competence using different techniques. Ligand/receptor supplementation has been recently studied as a method to delay spontaneous maturation in vitro to better mimic in vivo conditions. It was found that supplementing culture media with the physiologic ligand/receptor reagents C-type natriuretic peptide (CNP), estradiol, follicle stimulating hormone, and bone morphogenic protein 15 (BMP15) before standard IVM delayed maturation and improved oocyte developmental competence [39]. Cyclic AMP modulation has been the major focus for improving the IVM system. Cyclic AMP modulation has been implemented in different studies to either prevent the spontaneous drop in cAMP (phosphodiesterase (PDE) inhibitors) or stimulate the production of cAMP (adenylate cyclase activators) [40]. When used in culture before standard IVM, adenylate cyclase activators, and/or phosphodiesterase inhibitors have been shown to delay maturation and improve embryo development [41–45]. Cyclic AMP modulation in culture has also been implemented in a proposed culture system termed simulated physiological oocyte maturation (SPOM) which aims to mimic in vivo maturation conditions in vitro to generate better developmental competence for clinical applications such as infertility management, though results have varied by lab and species [46, 47]. We found the inhibition on PANX1 channels during maturation resulted in significantly higher cAMP concentrations following 6 h of maturation. Consistent with this finding, we found at 6 h of maturation a significantly higher number of treated oocytes remained at the GV stage. This suggests that inhibiting the PANX1 channels delayed maturation though it appears temporary as by 22 h a similar level of cAMP and number of mature oocytes were seen. Though, this delay appears to benefit the developmental competence of the oocytes as a higher proportion of treated oocytes successfully cleaved and developed to the blastocyst stage following maturation in the presence of 10Panx. Further evidence of delayed maturation can be seen following treatment with cAMP modulators. Treatment of COCs with cAMP modulators during IVM lengthens that time of cumulus-oocyte gap junctional communication [48, 49]. Our findings that cumulus expansion is decreased with 10Panx treatment suggests prolonged gap junctional communication similar to that during cAMP modification since cumulus expansion is a signal for gap junction communication loss [50]. Pannexins have been shown to play a major role as a conduit for the release of ATP by cells to the extracellular environment [51–53]. Interestingly, the activation of PANX1 hemichannels has been shown to result in higher cellular cAMP levels, in some cell types, as ATP has been shown to activate P2RY11 receptors [51, 54]. Conversely, the release of ATP through pannexin channels has been shown to result in decreased cellular cAMP concentrations [52]. This leaves two possibilities for the results we observed: One that the blockage of PANX1 channels decreases ATP release and results in a maintenance of cAMP levels in the oocyte delaying meiotic progression. Or two, that the PANX1 channels are playing a role in the programmed cumulus cell death occurring during meiosis and leading to decoupling of the cumulus cells from the oocyte. This is consistent with studies showing ATP release results in increased cellular cAMP levels and the activation of Caspase-3 [53, 55]. The blockage of 10PANX may limit the release of ATP preventing the programmed death of the cumulus cells and prolonging the maturation process. This is consistent with the delayed cumulus expansion we observed. Which mechanism is responsible for the delay in meiotic progression observed, following inhibition of the PANX1 channels, remains the subject of future research. These studies help support that PANX1 channels play a role in oocyte maturation; in that inhibition of these channels causes a delay in maturation and improved developmental competence by way of cAMP modulation and ATP release.

Finally, to determine a potential functional result to impairing PANX1 channel function we compared the level of ROS in the oocytes following maturation with exposure to 10Panx. We found that oocytes matured while inhibiting PANX1 channels had significantly less ROS when compared to our untreated controls. It has been well established that ROS buildup in oocytes is detrimental to continued embryo development. ROS have been implicated in causing negative developmental outcomes in embryos in vitro [56, 57]. ROS cause many different types of damage to developing embryos including a rise in lipid peroxides, increase in protein oxidation, and DNA strand breaks [56, 58, 59]. It has been shown that high levels of ROS in the in vitro culture environment are detrimental to bovine embryo development [60]. A more recent study by Li et al. showed the effects of cAMP modulation during IVM on ROS defense. They found that implementing a pre-IVM culture period with forskolin (an adenylate cyclase activator) and IBMX (PDE inhibitor) not only delayed maturation and improved embryo developmental outcomes, but also increased the levels of antioxidant glutathione (GSH) and decreased the intra-oocyte concentration of the ROS hydrogen peroxide [41]. This study could lead to experiments that connect inhibited PANX1 channels during IVM and cAMP modulation, with improved in vitro oocyte developmental competence and lower ROS.

In conclusion, we have found that PANX1 is expressed in bovine cumulus cells. It is also expressed at higher levels in the cumulus cells of less developed oocytes. PANX1 channel inhibition during IVM leads to a delay in meiotic maturation and greater developmental competence following IVF. Functionally, PANX1 inhibition during IVM potentially delays maturation by maintaining elevated cAMP levels, keeping spontaneous maturation from occurring as quickly, which in turn can lead to improved developmental outcomes. ROS levels were also decreased in the oocyte with PANX1 inhibition. These findings support that PANX1 channels are important in oocyte maturation and development and can be manipulated to provide better developmental outcomes during in vitro settings.

## Methods

All reagents were purchased from VWR (Radnor, PA) unless otherwise noted.

### In vitro maturation and in vitro fertilization

COCs were collected from abattoir-sourced, mixed breed *Bos taurus* ovaries. COCs underwent maturation and IVF followed by culturing to the blastocyst stage as previously described [61], with modifications. Briefly, follicles ranging from 2 to 5 mm were aspirated using a 10 ml syringe and 18-gauge, short-beveled needle. COCs, granulosa cells, and follicular fluid were collected into 50 ml conical tubes at room temperature. COCs with a uniform cytoplasm and at least three layers of cumulus cells were collected into TCM-199 supplemented with 8.3 mM sodium bicarbonate, 20 mM Hepes, 10% fetal bovine serum, 50 μg/ml gentamicine, and 22 μg/ml pyruvate. COCs were matured in TCM-199 supplemented with 8.3 mM sodium bicarbonate supplemented with 10% FBS, 50 μg/ml gentamicine, 22 μg/ml pyruvate, 1.1 mM glutamine, and 10 ng/ml EGF. Selected COCs were placed in groups of 20–25 per 90 μl microdrops consisting of oocyte maturation media either supplemented with 100 μM 10Panx (Tocris, Bristol, United Kingdom) or the vehicle only, under mineral oil (Sigma Life Science, Darmstadt, Germany) and cultured for 22 h. COCs were then used for experiments or fertilized in groups of 200 in 1.9 ml of fertilization media containing 10^6^ spermatozoa/ml for 18–22 h. To eliminate variation due to the male component, semen from a single ejaculation from a bull with proven in vitro fertility was used. Fertilization media consists of synthetic oviductal fluid (SOF, 1.17 mM CaCl_2_2H_2_O, 0.49 mM MgCl_2_6H_2_O, 1.19 mM KH_2_PO_4_, 7.16 mM KCl, 107.7 mM NaCl, 25.07 mM NaHCO_3_, and 5.3 mM Na-lactate) supplemented with 50 μg/ml gentamicine, 22 μg/ml pyruvate, 10 μg/ml heparin, 194.2 μg/ml caffeine, and 6 mg/ml BSA fraction V essentially fatty acid free (EFAF). Following fertilization, the presumptive zygotes were denuded by gently pipetting them up and down with a glass pipette. The presumptive zygotes were then cultured in groups of 25 in 50 μl culture media under mineral oil. Culture media consisted of 5 ml SOF supplemented with 6 mg/ml BSA fraction V EFAF, 1.1 mM glutamine, 2.8 mM myo-inositol, 0.57 mM sodium citrate, 22 μg/ml pyruvate, 50 μg/ml gentamicin, 1X essential amino acids, and 1X nonessential amino acids. Presumptive zygotes were then cultured in 5% CO_2_, 5% O_2_, and 90% N_2_ at 38.5 °C. Forty-eight hours after fertilization, cleavage rates were recorded based on the initial total number of oocytes. On day 7 after fertilization, morula/blastocyst rates were recorded based on the initial total number of oocytes. COCs in all experiments in this study were cultured at 38.5 °C and 5.0% CO_2_. All experiments were repeated at least 3 times.

### Immunocytochemistry of cumulus-oocyte complexes

COCs were aspirated from bovine ovarian follicles using a syringe and 18 gauge needle. This was followed by their collection under a stereoscope and washing in TCM199. Selected COCs were washed in phosphate buffered saline (PBS) then fixed for 15 min in 4% paraformaldehyde (PFA), blocked 30 min with PBS containing 3% bovine serum albumin (BSA) and 0.5% Triton-X, incubated overnight with a primary antibody (1:500 Rabbit anti-PANX1 CT-412; Western University, London, Ontario [62, 63]) (Supplementary Table 1), washed with blocking solution, and incubated 1 h with a secondary antibody (1:500, Goat anti-Rabbit FITC; ImmunoReagents Inc., Raleigh, NC) (Supplementary Table 1). Cells were counterstained with Hoechst 33342. A Nikon A1 Confocal Scanning Laser Microscope (Nikon Instruments, Melville, NY) was used to randomly image a single plane of the cumulus cells with or without the oocyte present. The anti-PANX1 CT-412 antibody was produced using a human epitope that has 86.67% homology with the bovine protein (unpublished results) [62].

### Brilliant cresyl blue staining of oocytes

Oocytes were aspirated from bovine ovarian follicles ranging from 2 to 5 mm in size using a syringe and 18-gauge needle. This was followed by their collection under a stereoscope and washing in TCM199. Oocytes with at least three continuous layers of surrounding cumulus cells and a homogeneous cytoplasm were then selected for brilliant cresyl blue (BCB) staining. Selected COCs were then washed three times in TCM199 and cultured 90 mins in TCM199 supplemented with 26 μM BCB. After staining COCs were washed in PBS and examined under a stereoscope. They were classified into different groups, high and low, according to the level of BCB coloration in the ooplasm. The categorized COCs were then pelleted and resuspended in radioimmunoprecipitation assay (RIPA) Lysis and Extraction buffer containing proteinase inhibitors.

### Western blotting

COCs or follicular fluid content were washed in PBS, pelleted, and frozen at − 80 degrees Celsius. When selecting for different antral follicle stages of development, follicles were aspirated separately from small antral (< 2 mm) and large antral (> 5 mm) follicles. Protein from the frozen samples was isolated using RIPA Lysis and Extraction buffer supplemented with protease inhibitors. Protein samples were quantified using a Qubit fluorometer (ThermoFisher, Waltham, MA). Samples were combined with loading dye, heated at 95 °C for 5 min, and ran through a 10% sodium dodecyl sulfate polyacrylamide (SDS) page gel, followed by transfer to a polyvinylidene difluoride (PVDF) membrane (Invitrogen, Carlsbad, CA). The membrane was blocked with tris-buffered saline (TBS) containing 5% BSA fraction V, washed with TBS containing 0.5% Tween-20, incubated overnight with primary, washed with TBS-Tween, incubated 1 h with a secondary antibody, washed with TBS-Tween, and exposed with enhanced chemiluminescence (ECL) for 4 min followed by chemiluminescent detection on an Azure c400 (Azure Biosystems, Dublin, CA). For PANX1 staining, a rabbit anti-PANX1 CT-412 (1:1 K, Western University, London, Ontario [61–63]) (Supplementary Table 1) primary and goat anti-rabbit horseradish peroxidase (HRP) (1:2 K, Columbia Biosciences, Frederick, MD) (Supplementary Table 1) secondary were used. GAPDH staining used a mouse anti-GAPDH (1:1 K, Sigma Life Science) (Supplementary Table 1) primary and a goat anti-mouse HRP (1:10 K, Novagen, Darmstadt, Germany) (Supplementary Table 1) secondary. The blots were densitometry analyzed and normalized to GAPDH using ImageJ analysis software. Tissues were derived from bovine samples.

### Propidium iodide dye uptake

Dye uptake studies were performed on bovine granulosa cells derived from follicular fluid collections (described above). Granulosa cells were collected from the follicular fluid by pelleting the cell content and washing with Dulbecco Modified Eagle Medium: Nutrient Mixture F-12 (DMEM/F12) media (D-MEM/F12 base powder (Gibco, ThermoFisher Scientific, Waltham, MA), 0.24% sodium bicarbonate). Isolated granulosa were plated in 6-well culture plates and incubated at 38.5 °C with 5% CO_2_. The media was changed every 24 h. 10Panx (100 μM) or vehicle control was added to the appropriate wells following 48 h of culture and incubated at 38.5 °C and 5% CO_2_ for 30 min. A propidium iodide (PI) dye uptake protocol was utilized using methods previously described [64], with modifications. Basal dye uptake was measured by adding PI (1 mM) to all wells and placing the dish back into the incubator for 30 min. Cells were then washed in PBS and fixed for 25 min in 4% PFA protected from light. Cells were washed with 3% PBS-BSA and nuclei were stained with NucBlue Live Cell Stain Hoechst33342 (Life Technologies, ThermoFisher Scientific, Waltham, MA) for 10 min protected from light. Images were captured using an Evos FL Cell Imaging System (ThermoFisher Scientific, Waltham, MA) using the RFP fluorescent filter, and PI positive cells quantified using ImageJ software. Three repetitions were conducted, and counting was performed by the same researcher for each repetition.

### Cumulus expansion measurement

To determine the expansion distance of the cumulus around the maturing oocyte, COCs were matured in groups of ~ 20–25 with or without 10Panx treatment (100 μM) for 22 h using methods described above. Culture plates with droplets were removed from incubation and and bright light images were taken using an Evos FL Cell Imaging System (ThermoFisher Scientific, Waltham, MA). Only completely visible COCs, where the distance from the center of the oocyte to the edge of the expanded cumulus could be measured, were included. Three repetitions were conducted with a total of *n* = 84 and *n* = 82 COCs for control and 10Panx groups respectively. ImageJ analysis software was used for measurements.

### Meiotic maturation of denuded oocytes

To determine the stage of nuclear maturation, COCs were matured with 10Panx treatment (100 μM) or vehicle control for 6 or 22 h using the previously described methods. Each treatment group and time point was removed from incubation when appropriate, washed in PBS, and placed in 0.25% trypsin for 10 min at 38.5 °C. The trypsinized COCs were then vortexed and denuded mechanically using a glass pipet. Denuded oocytes were selected and washed in PBS, then fixed in 4% PFA for 15 min. Fixed oocytes were permeabilized in 0.5% PBS-Triton for 15 min, washed in 3% PBS-BSA, and the nuclei were stained with NucBlue Live Cell Stain Hoechst 33342 (Life Technologies, ThermoFisher Scientific, Waltham, MA) for 10 min protected from light. Stained oocytes were mounted with coverslips on Superfrost microscope slides (Fisher Scientific, ThermoFisher Scientific, Waltham, MA) using VectaShield antifade mounting medium (Vector Laboratories, Burlingame, CA). Oocytes nuclear maturation state was recorded using the Evos FL Cell Imaging System (ThermoFisher Scientific, Waltham, MA) set to the DAPI fluorescent filter. Oocytes were classified as being in the germinal vesicle (GV), germinal vesicle breakdown (GVBD), or meiosis II (MII) stage of DNA meiotic maturation. Four repetitions were conducted with *n* = 73, *n* = 93, *n* = 120, and *n* = 114 oocytes for 6 h control, 6 h 10Panx, 22 h control, and 22 h 10Panx groups respectively.

### Direct cyclic adenosine monophosphate enzyme-linked immunosorbent assay

To determine the intercellular concentration of 3′,5′-cyclic adenosine monophosphate (icAMP) in COCs, COCs were matured with or without 10Panx treatment (100 μM) for 0, 3, 6, or 22 h using methods described above. Each treatment group and time point was removed from incubation when appropriate, and placed into maturation media supplemented with 0.5 mM 3-isobutyl-1-methylxanthine (IBMX). COCs were then washed again in maturation media supplemented with 0.5 mM IBMX, followed by a wash in maturation media without IBMX. The COCs were moved to PBS and then snap frozen in a 1.7 ml conical tube. Samples were stored at − 80 °C until the assay. The icAMP concentration of the COCs was determined using the Enzo Direct cAMP enzyme-linked immunosorbent assay (ELISA) kit (Enzo Life Sciences, Farmingdale, NY, Cat# ADI-900-066) using the acetylation protocol according to the manufacturer’s instructions. Optical density was then measured at 405 nm using an EMax Plus Microplate Reader (Molecular Devices, San Jose, CA) and icAMP concentration was calculated. Three experimental replicates were repeated with samples collected for each time point and treatment with *n* = 10 COCs per sample.

### 2′-7′-Dichlorodihydrofluorescein diacetate staining of oocytes

Following 22 h of maturation oocytes were mechanically denuded using a glass pipette. The denuded oocytes were then collected under a stereoscope and cultured for 30 min in PBS supplemented with 3% BSA, 10 μg/ml Hoechst 33342, and 10 μM 2′-7′-dichlorodihydrofluorescein diacetate (DCHF-DA). The stained oocytes were then washed briefly three times in PBS and immediately imaged for fluorescence under an Evos FL Cell Imaging System (ThermoFisher Scientific, Waltham, MA) using the DAPI and GFP fluorescent filters. Staining intensity was quantified using ImageJ analysis software by calculating the corrected total cell fluorescence (CTCF) of five areas of an individual oocyte and averaging them. The individual averaged CTCFs between 10Panx treated and vehicle only controls were then used to compare the DCFH-DA staining.

### Statistics

Statistical analysis was conducted using GraphPad Prism software (v6.01). Unpaired two-tailed Student t-tests were performed for all data sets besides meiotic maturation data, in which a one-way ANOVA was implemented per time point with Tukey’s post hoc testing for multiple comparisons. Data for PI dye uptake studies were log transformed for statistical analysis, however graphical representation is shown using non-transformed data. Data for developmental outcomes were arcsine transformed for statistical analysis, however graphical representation is shown with non-transformed data. Results are reported using standard deviation from the mean. Comparisons were considered statistically significant if *p* < 0.05 and contained a significant trend if *p* < 0.055.

## Supplementary information

**Additional file 1 Table S1.** Primary and secondary antibodies used for Western blotting and immunofluorescence.

## Data Availability

The datasets used and/or analyzed during the current study are available from the corresponding author on reasonable request.
