# Supplementary material for: Pannexin 1 inhibition delays maturation and improves development of Bos taurus oocytes
Source: J Ovarian Res. 2020 Aug 24;13:98. doi: 10.1186/s13048-020-00704-w (PMC7447567; doi:10.1186/s13048-020-00704-w)
Supplement: Supplementary file 1 — Additional file 1 Table S1. Primary and secondary antibodies used for Western blotting and immunofluorescence. [file 13048_2020_704_MOESM1_ESM.docx]

Supplementary Table 1

| Name | Dilution | Company/ Supplier | RRID |
| --- | --- | --- | --- |
| Rabbit anti-PANX1 CT-412 | 1:500 | Western University | See Materials and Methods or [38-39] |
| Mouse anti-GAPDH | 1:1000 | Sigma | AB_1078991 |
| Goat anti-rabbit FITC | 1:500 (IF) | Immuno Reagents, Inc. |  |
| Goat anti-rabbit HRP | 1:2000 (WB) | Columbia Biosciences |  |
| Goat anti-mouse HRP | 1:10,000 (WB) | Novagen |  |
